# History and development of trauma registry: lessons from developed to developing countries

**DOI:** 10.1186/1749-7922-1-32

**Published:** 2006-10-31

**Authors:** Benedict C Nwomeh, Wendi Lowell, Renae Kable, Kathy Haley, Emmanuel A Ameh

**Affiliations:** 1 The Department of Pediatric Surgery, Columbus Children's Hospital, The Ohio State University College of Medicine & Public Health, Columbus, OH, USA; 2 The Trauma Program, Columbus Children's Hospital, Columbus, OH, USA; 3 Division of Pediatric Surgery, Department of Surgery, Ahmadu Bello University and Ahmadu Bello University Teaching Hospital, Zaria, Nigeria

## Abstract

**Background:**

A trauma registry is an integral component of modern comprehensive trauma care systems. Trauma registries have not been established in most developing countries, and where they exist are often rudimentary and incomplete. This review describes the role of trauma registries in the care of the injured, and discusses how lessons from developed countries can be applied toward their design and implementation in developing countries.

**Methods:**

A detailed review of English-language articles on trauma registry was performed using MEDLINE and CINAHL. In addition, relevant articles from non-indexed journals were identified with Google Scholar.

**Results:**

The history and development of trauma registries and their role in modern trauma care are discussed. Drawing from past and current experience, guidelines for the design and implementation of trauma registries are given, with emphasis on technical and logistic factors peculiar to developing countries.

**Conclusion:**

Improvement in trauma care depends on the establishment of functioning trauma care systems, of which a trauma registry is a crucial component. Hospitals and governments in developing countries should be encouraged to establish trauma registries using proven cost-effective strategies.

## Background

A disease registry is a collection of uniform data describing individuals who meet specific inclusion criteria in which medical, demographic and other data are documented in an ongoing and systematic manner in order to serve predetermined purposes [1]. Disease registries are common in the United States (U.S.) and many other countries. Trauma registries are components of formal trauma systems at hospital, regional, state, and national levels.

Trauma registries evolved with the Quality Assurance (QA) movement, which is closely related to the twin concepts of Continuous Quality Improvement (CQI), or Total Quality Management (TQM). The essence of the QA philosophy is that the majority of defects in care results from failures of the system rather than the individuals themselves. The QA movement borrowed heavily from the Japanese industrial experience, in which a solid management philosophy was based on statistical process control. In this model, rigorous statistical methods were used to study industrial flow processes, ultimately leading to quality improvement [2]. With respect to trauma care, continuous, measurable improvement of care given to the injured patient is the goal of any QA system. A trauma registry is a timely, accurate, and comprehensive data source which allows for continuous monitoring of the process of injury care [3].

Successful deployment of trauma care systems, including the use of trauma registries, has played a significant role in the substantial decline in death and disability rates from injuries. Projections show that, between 2000 and 2020 road traffic deaths will increase by 83% in low- and middle-income countries. In contrast, there will be a further 30% decline in road traffic deaths in high-income countries, continuing a pattern that has been established in recent decades [4]. Without appropriate action, by 2020 road traffic injuries are predicted to become the third leading contributor to the global burden of disease worldwide and the second leading determinant of disability-adjusted life years (DALYs) in the developing countries [5].

Improvement in trauma care in Africa and other developing parts of the world will ultimately depend on the establishment of functioning trauma care systems, of which a trauma registry is a key infrastructural component. A trauma registry provides a means of collecting and analyzing pertinent epidemiologic data that can be used for the purposes of quality improvement, research, and planning. In preparing this report, the authors have drawn from their experience with an established trauma registry in a level-one pediatric trauma center in central Ohio, in the U.S., as well as ongoing efforts to initiate a trauma registry in northern Nigeria.

## Historical Perspective

The first computerized trauma database was established in 1969 at the Cook County Hospital, Chicago [6]. This registry became the prototype for the Illinois Trauma Registry, which began to accrue data from 50 designated trauma center hospitals across the state in 1971. Early registries were housed in a bulky mainframe computer, but in 1985 the first use of a microcomputer was reported [7]. Since then numerous hospitals, regions, states, and countries have developed trauma registries [8]. In the U.S. there are 37 states that maintain a trauma registry that include data on patients treated within trauma centers [9].

A well-designed trauma registry with validated and risk adjusted data can assist health care providers, legislators, and community health agencies in establishing a coordinated approach to trauma care. Although the primary source of trauma registry data is often a trauma center, such data is scalable to regional, state or national levels when individual registries adhere to a common set of standards. Reporting of trauma center data to a regional, state, and national trauma registry is an important function of an institutional registry. In some states, a designated authority requires submission of data to a higher level data repository; for other states submission is voluntary. Regardless whether a voluntary or mandated requirement, pooling of multi-center trauma data can be used for purposes ranging from epidemiologic reports to comparisons of trauma centers' effectiveness and to evaluation of performance improvement indicators.

The American College of Surgeons Committee on Trauma (ASCOT) commissioned the Major Trauma Outcome Study (MTOS) in 1982 to pool data on injured patients and to develop and test survival probability norms based on injury severity scores [10]. The MTOS data was collected retrospectively with four countries participating: the U.S., Canada, United Kingdom, and Australia. Survival probability norms were generated using the Revised Trauma Score (RTS), Injury Severity Score (ISS), patient's age, and injury mechanism (TRISS methodology). More recently, ASCOT has established the National Trauma Data Bank (NTDB), which collates prospective trauma registry data from trauma centers and trauma systems in the U.S. The NTDB is the largest aggregation of trauma registry data ever assembled and contains over one million records from 405 U.S. trauma centers. The information contained in the data bank has become a potent instrument in advancing trauma care in such areas as epidemiology, injury control, research, education, acute care, and resource allocation [11]. Similarly, the National Pediatric Trauma Registry (NPTR) was a model of collaboration among hospitals committed to improving care of injured children. Before project funding was terminated, the NPTR accrued over 100,000 cases between 1985 and 2003 from 80 participating hospitals, which reported their data on a voluntary basis [12].

## How Does a Registry Improve Trauma Care?

Trauma registries contribute to processes that improve care [13]. An integrated, concurrent trauma registry provides an ideal information system for a performance improvement process, which is an essential requirement for trauma centers and systems. The trauma registry serves as a repository for specific data that can be evaluated, trended, and linked to outcomes. In busy trauma centers and systems, the trauma registry serves as the hub for quality control queries.

The trauma registry supports the performance improvement process by serving as conduit to monitor trauma system trends, supplying benchmarking data, and identifying injury trends including distribution by age, geographic location, and cause of injury. It also generates data for the evaluation of outcomes for a specific trauma entity and provides information that can be used to evaluate timeliness, appropriateness, and quality of patient care. Of special importance is that the trauma registry supports a statistical model for the evaluation of trauma activity and facilitates trauma research endeavors. The NPTR, for example, was the basis for over 60 peer-reviewed publications and countless citations in a variety of publications including trauma practice guidelines and public policy statements [12]. The trauma registry can also be used to integrate financial data with care delivery data and to project resource utilization for trauma centers and systems. Finally, the trauma registries supply data relative to provider credentialing and provides information support for accreditation, verification, and designation processes.

## How to Design and Implement a Trauma Registry

A trauma registry typically includes detailed information about the cause, nature, and severity of the injury. However, trauma registries are highly variable in all aspects of their operational infrastructure. Most registries are limited to patients treated in trauma centers but exclude those who die at the scene or those with minor injuries who do not require hospital treatment. Often, they will include data on deaths occurring in both the emergency departments and following admission to the hospital. Some registries collect data only on "major" trauma patients and may exclude survivors who are released from the emergency department or who were admitted for less than three days [14].

While terminology for levels of trauma centers varies by region, generally trauma centers are described as a level 1, 2, 3, or 4 with 1 being the highest level. Each type of center corresponds with a level of resources for the trauma patient; this also carries over into requirements for research, prevention, and other support services. Variability among trauma centers and trauma data registries is significant. Variations may include (1) concurrent versus retrospective data collection, (2) expanded versus limited data element collection, (3) computer versus filed repository, (4) trauma trained registrars versus health information specialist as the collector, (5) linkage to performance improvement activities versus limited use of the data, (6) non validated data versus validated data, (7) small trauma census versus large trauma census, and (8) risk adjusted data versus expanded non-risk adjusted data.

In any setting, the essential operation of an effective trauma registry requires adequate funding, reasonable and dependable software, a well-defined patient population, adequately trained personnel, a process for data collection, reporting, and validation, and a process for ensuring privacy. Generally, trauma registries are maintained by trauma registrars and/or the equivalent of trauma registry coordinators. Beginning in 2000, a nationally recognized certification process for trauma registry personnel was initiated in the U.S., leading to the designation of CSTR (Certified Specialist in Trauma Registry). Prior to obtaining certification, the registrar must have at least two years experience in data management and have completed the appropriate courses. Essential training for trauma registrars includes software instruction, ICD-9 coding, Abbreviated Injury Scale (AIS) training, Basic and Advanced Registrar Courses, medical terminology, and anatomy courses.

Trauma registry courses and workshops are held periodically at regional and national trauma meetings. In 1988, a workshop was held under the auspices of the Centers for Disease Control (CDC) to develop standard case criteria and a uniform, minimum data set for trauma registries. The trauma registry workshop provided the first opportunity for a multidisciplinary group of physicians, researchers, public health officials, and health care administrators to participate in the formulation of standards for trauma registries [15,16]. Information on upcoming trauma registry courses is currently maintained by the American Trauma Society [17].

### Designing the Data Set

A good starting point in the design of a trauma database is to arrive at a definition of the *trauma patient*, which varies based upon local, regional, state or national guidelines. Included in the definition is consideration for age differentiation for pediatric and adult trauma patients. Patients are then identified as candidates for inclusion into the trauma registry by using specific inclusion criteria (specific rules allowing a patient to be included in the data collection system) or exclusion criteria (specific rules disallowing a patient to be included in the data collection system) to initiate the data collection. Many trauma registries have developed more specific inclusion or exclusion criteria that are used to define the trauma patient captured in a registry. For example, in Ohio the trauma patient is defined as one who is at significant risk for loss of life or limb, or significant permanent disfigurement or disability from a blunt or penetrating injury, exposure to electromagnetic, chemical, or radioactive energy, drowning, suffocation, or strangulation, or a deficit or excess of heat.

For an institution beginning to collect trauma data, consideration of regional, state, and national inclusion requirements are important factors. In the U.S., the national consensus for inclusion criteria in trauma registries is ICD-9-CM 800 – 959.9. A sample of the Ohio trauma registry inclusion and exclusion criteria is described in Tables 1 and 2. Trauma registry protocols are inconsistent across centers and the inclusion and exclusion criteria vary widely [18,19]. It is important that the exclusion criteria be chosen carefully because even slight variations may considerably alter the apparent severity of injury and the resource utilization they portray thus diminishing the representativeness of the data set [19].

**Table 1:** Ohio Trauma Registry Patient Inclusion Criteria*

**Category A: *One of the following***			
1. Patient's first or initial admission for at least 48 hours			
2. Patients who transfer into or out of any hospital, regardless of their length of stay			
3. Patients who are dead on arrival (DOA)			
4. Patients who die after receiving any evaluation or treatment while on hospital premises			

**Category B: *One of the following diagnoses***			

**ICD-9-CM Diagnosis Codes**	**ICD-9-CM Diagnoses Descriptions**		

800.0 – 819.9	Fractures		
821.0 – 904.9	Fractures, dislocations/sprains, intracranial injury, internal injury of thorax, abdomen and pelvis, open wounds, injury to blood vessels		
911.0, 911.1, 912.0, 912.1	Abrasions or friction burns to trunk, shoulder and upper arm		
916.0, 916.1, 919.0, 919.1	Abrasions or friction burns to hip, thigh, leg, ankle, other or multiple sites		
920.0 – 929.9	Contusions and crush injury		
940.0 – 959.9	Burns, injury to nerves and spinal cord, traumatic complications and unspecified injury		
991.0 – 991.6	Frostbite, hypothermia and external effects of cold		
994.0, 994.1, 994.7, 994.8	Asphyxiation, strangulation, drowning, and electrocution		
987.9	Smoke inhalation		
995.50 – 995.59	Child maltreatment and abuse		

***OR***			

**ICD-9-CM Diagnoses**			**E-CODE**

348.4	Uncal herniation	**AND WITH**	
348.5	Cerebral Edema	**ANY OF THE**	E800 – E848.8
348.8	Pneumocephalus	**FOLLOWING**	E877.8 – E905.0
372.72	Subconjunctival hemorrhage	**External Cause Codes**	E906.0 – E928.8
518.5	Traumatic ARDS	**(E-Codes)**	E950.0 – E999
784.7	Epistaxis		

Codes separated by a hyphen indicate a range of codes including both codes AND all codes in between. Example 800.0 – 801.5 Codes separated by a comma indicate a single code. Example 901.1, 901.2, 901.8

*Adapted from Ohio Trauma Registry Data Element Description Manual, January 2003 edition, Division of EMS, Ohio Department of Public Safety.

**Table 2:** Ohio Trauma Registry Patient Exclusion Criteria*

**ICD-9-CM Diagnoses Codes EXCLUDED**	
820.0 – 820.9	Isolated hip fracture
905 – 909	Late effects of injury
910.0 – 910.9, 911.2 – 911.7, 912.0 – 918.9, 919.2 – 919.7	Superficial abrasions, blisters, insect bites
930 – 939	Foreign bodies

**External Cause Codes EXCLUDED**	

E849 – 849.9	Place of occurrence
E850 – E869.9	Poisonings
E870 – E876	Misadventures during surgical and medical care
E905 – E905.9	Venomous animals and plants (except snakes)
E929 – E929.9	Late effects of accidental injury
E930 – E949	Drugs, medicinal & biological substances, causing adverse effects in therapeutic use

*Adapted from Ohio Trauma Registry Data Element Description Manual, January 2003 edition, Division of EMS, Ohio Department of Public Safety.

The next task is to design a data set, which is valid, reliable, and efficient to collect. Careful selection and definition of each data point is essential for the success of any database [3]. Too little data would have limited value, but too much data could be time-consuming and expensive to collect and administer. In order to facilitate rapid retrieval and analysis of data it is necessary to assign codes to the injury event, the treatment, and the outcomes. It is valuable to adopt a coding system that is compatible with national or international norms to allow for easy comparison of data. In addition to demographic details, most registries include information on the injury mechanism, vital signs, laboratory and radiologic testing, initial and definitive treatment procedures, utilization of hospital resources, and patient outcome. A measure of injury severity is usually included, such as the Glasgow Coma Score (GCS), Revised Trauma Score (RTS), and the Injury Severity Score (ISS). Another common practice is the calculation of the probability of survival based on the Trauma and Injury Severity Scoring (TRISS) method. While the use of these severity scores and outcome predictive criteria can facilitate comparison with national and international norms, they have significant limitations with respect to interobserver reliability, validity, and interpretation [20-25]. Furthermore, instruments that were developed in Europe and North America have not always proven reliable elsewhere [21,26-30]. Scarce resources limit the practical application of these instruments in developing countries, creating the need for innovative methods tailored to the needs of developing countries. One such novel system is the Kampala Trauma Score (KTS), which is a simplified composite of the RTS and the ISS and closely resembles the TRISS method. The validity of the KTS was demonstrated when compared with RTS and ISS alone, or compared with the TRISS method [31]. The KTS has proven reliable when used in trauma registries in Uganda, both in urban and rural settings [32-34].

### Computer Hardware and Software

There is great variability in the design of trauma databases. Initiation of a trauma registry should include technical consideration for operating systems such as hardware, software, operating systems, memory support, and security.

The initial design of a trauma database should include expert advice from information technologists. Some institutions invest in consulting services and others may be required to explore commercial systems due to local and national standards. Trauma databases may be expensive endeavors and without a well-designed infrastructure may yield an ineffective repository for the original objectives. More commonly used registry software packages in the U.S. are TraumaBase™ (Clinical Data Management, Inc., Conifer, CO), Trauma One™ (Lancet Technology, Inc., Boston, MA), Trauma!™ (Cales and Associates, LLC, Louisville, KY), Collector™ (Digital Innovation, Inc., Forest Hill, MD), and NATIONAL TRACS™ (American College of Surgeons, Chicago, IL). Many of these programs can run on standalone PC's using a Microsoft Windows XP™ or Win2000™ platform, network workstation with the Windows NT™ operating system, web-enabled systems, and even DOS-based systems. A Macintosh™ version is available for some of these programs as well as those capable of utilizing the LINUX™ operating system. A network linkage enables multiple users, greater access, and a built-in back-up system.

Hardware requirements are similar across different software packages. For example, a standalone PC running the TraumaBase™ requires a minimum of an Intel Pentium™ class (or similar) processor, CD-ROM Drive, VGA Monitor, 256 Megabytes of Memory, 120 Megabytes of hard disk space plus an additional 2 Kilobytes per patient. Internet access using a 56 k (or higher) modem is desirable for online technical support. Additional software may be required for optimal implementation.

The computer hard disk storage capacity needs to match the needs of a growing program. As a rule of thumb, one should allow approximately 200 megabytes (MB) for program installation plus 2 kilobytes (KB) per patient. For example, a registry with 1000 patients per year over a period of 10 years would require a minimum hard disk space of 220 MB. More storage will be needed if backups or archives will be kept on the computer hard disk. Hard disks with 20 gigabyte (GB) or more capacity have more than enough room for a trauma registry and for word processors, spreadsheets, or other useful programs.

Random Access Memory (RAM) capability should be at least 32 MB (the more the better) running at the highest processor speed (i.e., 1 gigahertz or greater) available. It is quite common to find reasonably priced computers with 512–1024 MB of RAM running at 2–3 gigahertz with ≥80 GB hard disk capacity.

Security of a database is a complex issue requiring dedicated technical expertise. In the U.S., hospital and federal standards typically guide internal policies and procedures. One of the most common ways to protect data is using a password. Some databases are more sophisticated and allow for access levels within the software itself. With the technology advancements in storage chips, many computers can house large databases. It is an industry recommendation to back up computer files every day. With large files and databases there are varieties of storage media such as diskette, tape, compact disc (CD), digital video disc (DVD), and flash drives. Even on network systems, all computers should be backed up frequently. Most systems will perform a back up nightly because of the length of time required to do this [35]. Although registries may begin small or abbreviated, planning for expansion over many years duration is recommended.

Ideally, a trauma registry requires an uninterrupted power supply, a condition unattainable in most developing countries. In order to prevent costly loss of data, investment in back-up power technology is highly desirable. Portable computer platforms may prove more expedient in an environment that cannot support reliable operation of a trauma registry that depends on fixed computer equipment. A mobile trauma registry using a handheld computer was successfully implemented under the hostile combat conditions of the Operation Enduring Freedom in Afghanistan [36].

## Experience with Trauma Registries in Developing Countries

Trauma registries in most developing countries either do not exist at all (particularly in sub-Saharan Africa) or where they exist, are often rudimentary, poorly developed and incomplete. Much of the data on the epidemiology of trauma from developing countries are one-time surveys, retrospective clinical studies, mortuary data or population surveys [37-39]. Periodic population-based surveys are limited by recall and do not provide adequate information on individual patients and injury severity.

A well-designed trauma registry is desirable in developing countries because it can make significant positive impact on the implementation, evaluation, and planning for trauma care. However, because of the stiff competition for healthcare dollars, innovative registries must be designed that do not depend on costly infrastructure and highly trained technical manpower. At present, several factors exist in many developing countries that hamper the establishment of reliable and efficient trauma registry (Table 3). Fortunately, there is increasing interest in the use of trauma registries in many developing countries and with successful outcomes.

**Table 3:** Barriers to efficient trauma registry in developing countries

• Little or no pre-hospital care	
• Non-availability of (or inefficient) evacuation and transportation system	
• Limited inter-hospital communication in case of transfers	
• Lack of standardized and uniform hospital data formats	
• Limited availability of electronic data storage and retrieval facilities	
• Inadequate funding	
• Unfavorable government health policies	
• Inadequate census and population data	
• Lack of awareness in the communities	

At a major university trauma center in Karachi, Pakistan, a daily log of all trauma admissions, transfers, or deaths were maintained using the emergency room register. Data acquired during initial assessment of patients were used to calculate the probability of survival based on the TRISS method. The data was subsequently entered into public-use trauma registry software provided by the CDC [26]. This registry has also been used to establish a basis for local peer review audit of trauma deaths and in comparing outcomes between hospitals [26,40].

A more simplified system was established in Uganda, where a hospital-based registry was initiated as the first step in establishing an injury surveillance system [33]. This program utilized a minimal data set in the Mulago and Kawolo hospitals, where trained staff used a one-page, 19-item registry form to collect data on demographics, injury causation, and outcome. Funded by the United States Agency for International Development (USAID), the study demonstrated the feasibility of establishing an effective trauma registry in an urban and rural setting using limited resources and without a sophisticated software package. This program has been successfully extended to five large hospitals (60 – 1,200 beds) in Kampala and also Addis Ababa, Ethiopia [34,41]. A major outcome of this effort was the demonstration of the utility of the KTS as a triage tool in settings where limited resources preclude the use of injury measures developed in western countries.

Over the past 5 years, there have been ongoing efforts to develop and establish a trauma registry in a large teaching hospital in northern Nigeria. The first phase of this program has been implemented and is limited to injured children. The registry utilizes a simple 10-point data set in a single sheet, including demographics, mechanism and severity of injury, treatment and outcome. It includes only children whose injuries are severe enough to warrant hospital admission. Migration to an electronic database is pending availability of funds. In the second phase, the registry will be expanded to include adult patients and also other regional hospitals. However, as in many developing countries, the effort is being hampered by several difficulties (Table 3), particularly scarcity of funds and lack of adequately trained staff. Nonetheless, the registry has helped in the re-organization of pediatric trauma care in this hospital.

## Limitations of a Trauma Registry

There are certain drawbacks of a trauma registry that limit its value as a tool for public health surveillance. Many injured patients in developing countries either do not survive to reach a hospital or do not seek formal hospital treatment and are not captured by hospital-based registries. Some registries also exclude patients who do not rigidly fall into their defined criteria for *major trauma*. Current hospital-based registries are therefore not representative of all injuries in the population. Differences in case criteria and data contents, concerns about completeness and quality, and the limit in geographic and population coverage, limit their value for quality control, injury surveillance, and planning for health services [42].

The cost implications of implementing a trauma registry under conditions of limited healthcare budgets must be carefully considered. The benefits must be weighed against the costs in terms of infrastructure and work force. Examples from the few developing countries discussed earlier show that trauma registries can be implemented in a cost-effective manner. A suggested guide to developing a trauma registry in developing countries is summarized in Figure 1.

## Conclusion

Databases have been described as the engine of change in today's healthcare environment, especially in the trauma center [35]. Therefore any hospital that cares for injured patients can benefit from some process of trauma data management. A trauma registry serves as a conduit for trauma data that drives the evaluation, prevention, and research of trauma care and can be used for quality control and planning. For this reason, hospitals and governments in developing counties should be encouraged to participate in trauma registries and other health information management systems.

## Competing interests

The author(s) declare that they have no competing interests.

## Authors' contributions

Conception: BCN, EAA

Drafting of manuscript: BCN, WL

Critical revision: RK, KH, EAA
